# Characterization of increased cuticular wax mutant and analysis of genes involved in wax biosynthesis in *Dianthus spiculifolius*

**DOI:** 10.1038/s41438-018-0044-z

**Published:** 2018-08-01

**Authors:** Aimin Zhou, Enhui Liu, Jiao Liu, Shuang Feng, Shufang Gong, Jingang Wang

**Affiliations:** 1 College of Horticulture and Landscape Architecture, Northeast Agricultural University, Harbin, 150030 China; 2 Key Laboratory of Saline-Alkali Vegetation Ecology Restoration in Oil Field (SAVER), Ministry of Education, Alkali Soil Natural Environmental Science Center (ASNESC), Northeast Forestry University, Harbin, 150040 China

## Abstract

Cuticular wax formation on the surface of plant leaves is associated with drought-stress tolerance. The identification of wax biosynthesis-related genes will contribute to the genetic improvement of drought resistance in plants. In this study, we characterize a novel *Dianthus spiculifolius* mutant with increased cuticular wax. The mutant exhibited stronger drought resistance as indicated by less leaf wilting and death, higher leaf relative water content and water retention capacity, and slower water loss and chlorophyll extraction than did the wild type during drought treatment. In the mutant leaves, 2 730 upregulated and 2 151 downregulated differentially expressed genes (DEGs) were identified by transcriptome sequencing. A wax biosynthesis pathway of the identified DEGs was significantly enriched. Finally, three key genes (*DsCER1*, *DsMAH1*, and *DsWSD1*) involved in wax biosynthesis were identified and verified by qPCR. These results suggest that differential expression of DEGs involved in wax biosynthesis may be associated with the increase in cuticular wax in the mutant. Taken together, our results help elucidate wax formation patterns in *D. spiculifolius*. Furthermore, the DEGs involved in wax biosynthesis identified here may be valuable genetic resources for improving plant stress tolerance through increased accumulation of cuticular wax.

## Genetics: Wax producing genes linked to drought resistance

Genetic analyses of a *Dianthus spiculifolius* mutant reveals three genes involved in cuticular wax production that could be crucial for conferring drought resistance. *D. spiculifolius* is a perennial herbaceous flowering plant that shows strong resistance to cold and drought stress. A study led by Shu-fang Gong and Jin-Gang Wang at Northeast Agricultural University in Harbin, China, examined a *D. spiculifolius* mutant with significantly more wax deposits on leaf surfaces and stronger drought resistance than wild-type plants during drought treatment. Three genes involved in the production of cuticular wax (*DsCER1*, *DsMAH1* and *DsWSD1)* were highly expressed in the mutant and associated with the leaves’ improved water holding capacity. Further understanding how plants adapt and protect themselves against water loss will help to produce genetically improved drought-resistant plants.

## Introduction

Drought stress, as one of the major environmental stress factors, seriously affects plant growth and development as well as crop production. Therefore, plants have evolved a variety of mechanisms to adapt to drought-stress conditions. At present, the mechanism of plant response to drought stress has been extensively studied in terms of morphology, physiology, gene expression, and molecular genetic studies^1^. Several direct mechanisms have been widely reported, such as modulation of stomatal movement, synthesis of osmoprotectants, and maintenance of root growth^2,3^. Understanding these mechanisms will contribute to the genetic improvement of drought resistance in plants.

Stomatal control of leaf transpiration is a primary defense mechanism that prevents stomatal water loss during drought stress^4^. Recent studies have reported the important role of cuticular wax accumulation in plant resistance to drought stress by regulating non-stomatal water loss^5–10^. Cuticular wax consists a complex mixture of very-long-chain fatty acids (VLCFAs, chain lengths ranging from C20 to C34) and their derivatives such as alkanes, primary and secondary alcohols, aldehydes, ketones, wax esters, and often triterpenoids and flavonoids^11^. Cuticular wax biosynthesis occurs mainly via either the decarbonylation or acyl-reduction pathway. The decarbonylation pathway results in the synthesis of aldehydes, alkanes, secondary alcohols, and ketones, whereas the acyl-reduction pathway yields primary alcohols and wax esters ^12,13^.

In *Arabidopsis*, a series of genes related to wax biosynthesis have been identified and characterized^14^. In the decarbonylation pathway, a multiprotein complex consisting of eceriferum (CER1/CER3) helps catalyze the conversion of VLC acyl coenzyme A to alkane^15^. Midchain alkane hydroxylase 1 (MAH1) converts an alkane produced from the VLCFAs into a secondary alcohol and subsequently oxidizes the secondary alcohol to a ketone^16^. In the acyl-reduction pathway, a primary alcohol is produced from the VLCFAs by fatty acyl coenzyme A reductase (FAR3/CER4), and may then be converted into wax esters by bifunctional wax ester synthase/diacylglycerol acyltransferase (WSD1)^17,18^. Understanding the wax biosynthesis pathway and the genetic mechanism of cuticular wax accumulation in high-wax-producing plants will contribute to the improvement of plant resistance to environmental stress by genetic manipulation.

*Dianthus spiculifolius* Schur, a perennial herbaceous flowering plant in the Caryophyllaceae family, exhibits strong resistance to drought stress^19^. Moreover, *D. spiculifolius* exhibits a number of important application characteristics, such as adaptability to a wide range of environments, strong resistance to trampling, and high ornamental value. It is projected to be an important lawn grass in the future. In the present study, in the M2 generation of ethyl methanesulfonate (EMS)-mutagenized *D. spiculifolius*, we obtained a high-wax-producing mutant, which was named ‘greyish-green’ (GG), according to the color of its leaf. In addition, we observed the crystal pattern of wax compounds on the leaf surfaces of GG *D. spiculifolius* mutants using scanning electron microscopy (SEM). Further, we compared the drought resistance of GG and wild-type (WT) *D. spiculifolius* plants. Moreover, we used transcriptome sequencing to screen for wax biosynthesis-related genes in GG plants. Finally, we identified three key wax biosynthesis genes and examined their expression patterns by quantitative real-time PCR (qPCR). Our results will help elucidate wax formation patterns in *D. spiculifolius*. Moreover, the identification of wax biosynthesis-related genes will provide a potentially valuable resource for genetic improvement of high-wax-producing plants.

## Materials and methods

### Plant growth conditions and drought treatment

*D. spiculifolius* plants (WT) were grown in greenhouses and in an open field at the Northeast Agricultural University (Harbin, China; 128.4° E, 45.0° N). M2 generation EMS-mutagenized *D. spiculifolius* plants (GG) were also used in this experiment. Potted plants were grown in a growth chamber under a 12/12-h light/dark photoperiod (20–40 μm sec^−1^ m^−2^ light intensity) at 22 °C.

For drought treatments, 3-month-old potted GG and WT plants were transferred to 30 °C, at which time watering was stopped, under the same light source. After 8 d of drought treatment, the GG and WT plants were re-irrigated for 4 d for recovery. Leaf samples at identical positions were collected for biochemical measurements after initiation of the stress treatment. Leaf sampling was repeated six times per treatment.

### **Scanning electron microscopy (SEM)**

For SEM analysis, leaf samples from GG and WT plants were collected and fixed with glutaraldehyde buffer, then gradually dehydrated by alcohol. The leaf samples were then dried to the critical point under liquid CO_2_ and sputter coated with an electrically conductive gold layer before being imaged by SEM (Hitachi SU-8010, Tokyo, Japan) at 5 kV.

For cryo-SEM analysis, leaf samples from GG and WT plants were sprinkled onto a perforated aluminum stub and plunged into liquid nitrogen slush (−210 °C). The frozen samples were transferred to a cryo system (PP3010T; Quorum Technologies, Lewes, UK), sputter coated with platinum, and transferred to the SEM cold stage and examined at −140 °C at a beam voltage of 5 kV and probe current of 10 mA.

### Water loss and chlorophyll leaching assays

Water loss and chlorophyll leaching assays were performed as previously described^5^. Three-month-old GG and WT plants grown under either normal and drought-stress conditions at different times were dark acclimated for 12 h to assure stomatal closure. The leaves were subjected to water loss measurements and chlorophyll leaching assays.

### RNA-seq and transcriptome data processing

Total RNA from the leaves of GG and WT plants was isolated using TRIzol reagent (Invitrogen, Carlsbad, CA, USA). The quality of the RNA samples was confirmed, and the samples were sent to the Beijing Genomic Institute (BGI, Shenzhen, China) for RNA sequencing. The RNA-seq read data (accession number SRP125917) were deposited in the NCBI Sequence Read Archive.

Transcriptome data processing was performed as previously described^19^. All assembled unigenes were compared with public protein databases, including the NR (NCBI non-redundant protein sequence), NT (NCBI nucleotide sequence), KEGG (Kyoto Encyclopedia of Genes and Genomes), Swiss-Prot protein, KOG (euKaryotic Ortholog Groups), InterPro, and GO (Gene Ontology) databases.

### Analysis of differentially expressed genes (DEGs)

Using the RPKM (reads per kilobase per million reads) method^20^, expression levels of the unigenes were calculated. The formula underlying the RPKM method was described by Zhou et al^19^. All DEGs were mapped to each term of the KEGG or GO databases, and significant pathways were defined based on a corrected *P* value ≤ 0.05. The *P* values were corrected for multiple testing using false discovery rate (FDR) methodology. DEGs were screened with an FDR threshold of 0.05 or less and an absolute log_2_ ratio of 1 or more.

### **qPCR expression analysis**

Expression levels of three key genes (*DsCER1*, CL588.Contig5; *DsWSD1*, CL538.Contig3; *DsMAH1*, Unigene7857) related to the wax biosynthesis pathway were investigated by qPCR. The reference sequence was *DsActin* (CL2553.Contig5). The primers used in this assay are shown in Supplementary Table S1. qPCR was carried out as described by Ren et al^21^. Three biological and three technical replicates were performed per sample.

### Measurement of physiological indexes

Measurements of relative water content (RWC) of leaves were performed as previously described^22^. Chlorophyll and carotenoid content were measured according to the method described by Parida et al^23^. These measurements were repeated six times.

### **Statistical Analysis**

All experiments were conducted at least in three independent biological and three technical replicates. The data were analyzed using one-way analyses of variance in SPSS (SPSS, Inc., Chicago, IL, USA), and statistically significant differences were calculated based on Student’s *t* test, with *P* *<* 0.05 (*) and *P* < 0.01 (**) as the thresholds for significance.

## Results

### **Phenotype and drought tolerance of*D. spiculifolius*cuticular wax mutants**

**Fig. 1:**
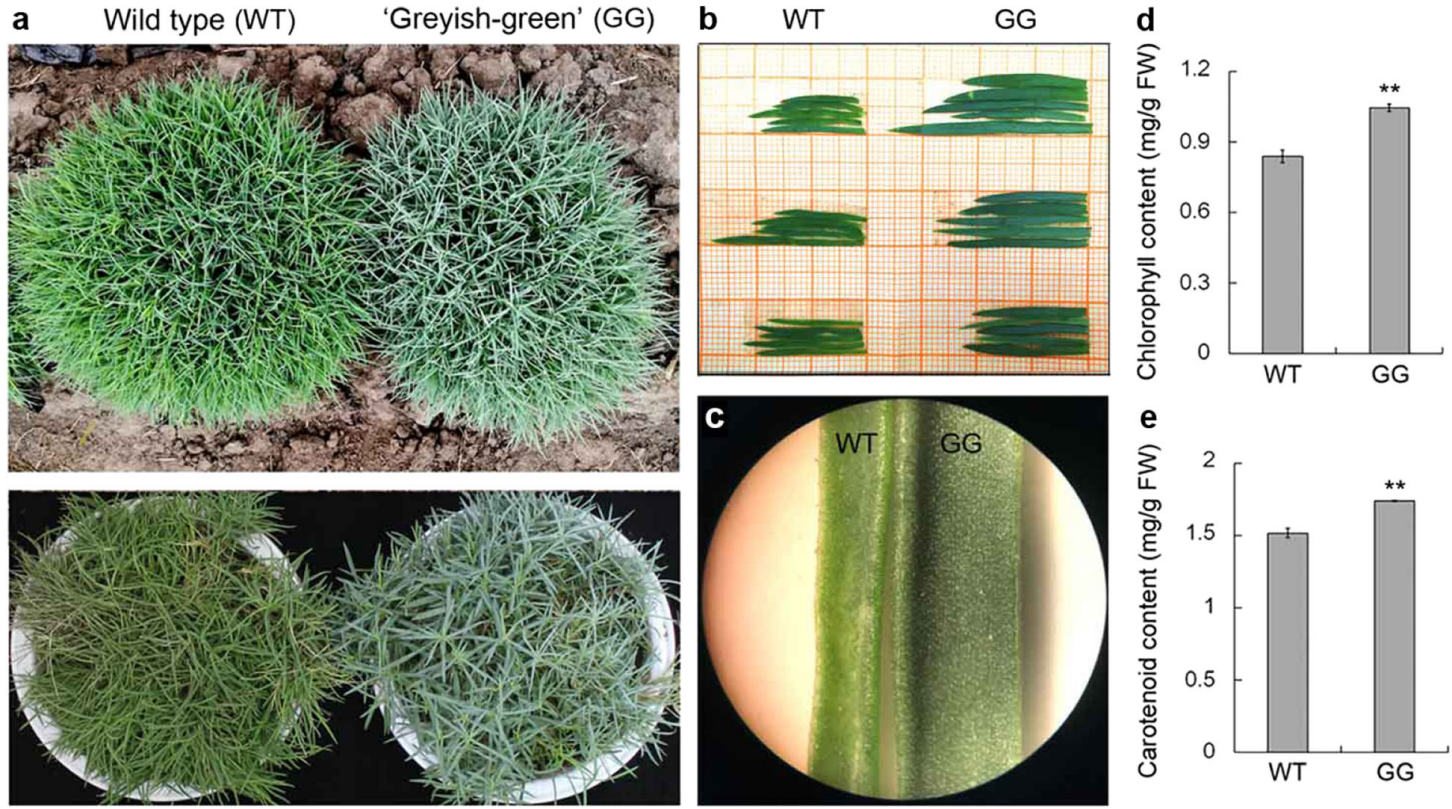
Phenotype of cuticular wax mutants of *D. spiculifolius*. **a** Wild type (WT) and cuticular wax mutants (‘Greyish-green, GG)’ of *D. spiculifolius* grown in an open field and a greenhouse, respectively. Leaf morphology (**b**) and color (**c**) of WT and GG plants. Comparison of chlorophyll (**d**) and carotenoid (**e**) content of WT and GG leaves. Asterisks indicate significant differences between WT and GG plants (**P < 0.01; Student’s *t* test). FW fresh weight

**Fig. 2:**
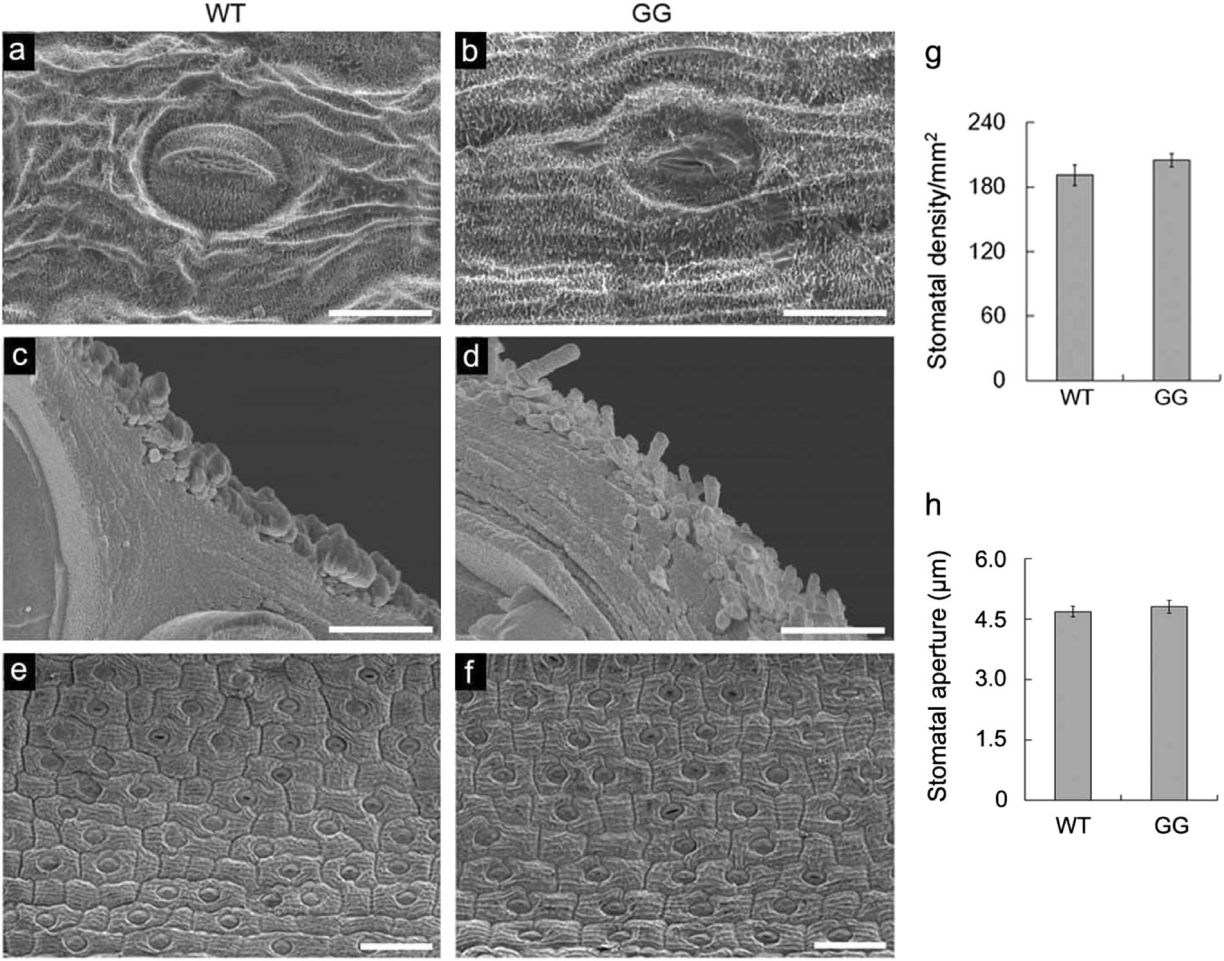
Scanning electronic microscopy (SEM) of WT and GG *D. spiculifolius* leaf surfaces. SEM images of the leaf surfaces (**a**, **b**, **e**, **f**) and leaf freeze-fracture cross-sections (**c**, **d**) of WT and GG plants. Comparison of stomatal density (**g**), and stomatal aperture (**h**) of WT and GG leaves. Asterisks indicate significant differences between WT and GG plants (**P < 0.01; Student’s *t* test). Error bars represent SD (*n* = 3). Scale bars = 20 μm (**a**, **b**), 5 μm (**c**, **d**), and 100 μm (**e**, **f**)

Clones of the GG plant propagated by stem cuttings had the same phenotype as that of the mother plant (Fig. 1a). The width and color of the leaves of the GG plants were different from those of the WT plant (Fig. 1b, c). Because leaf color was affected by pigment content, we first investigated the pigment content in leaves of GG and WT plants. Our results showed that chlorophyll and carotenoid contents were significantly higher in leaves of GG than in those of WT plants (Fig. 1d, e). In addition, we used SEM to examine differences in deposition on leaf surfaces. The number of rod-like wax crystals on the leaf surfaces of GG plants was much higher than that on the leaves of WT plants (Fig. 2a-d). Wax is readily soluble in organic solvents such as chloroform. Expectedly, these wax crystals dissolved readily after being immersed in a chloroform solution for 30 s (Supplementary Figure S1). These results indicate that the GG plant is an enhanced cuticular wax mutant. In addition, there was no difference between the stomatal density and aperture of GG and WT leaves (Fig. 2e, f, g, h).

**Fig. 3:**
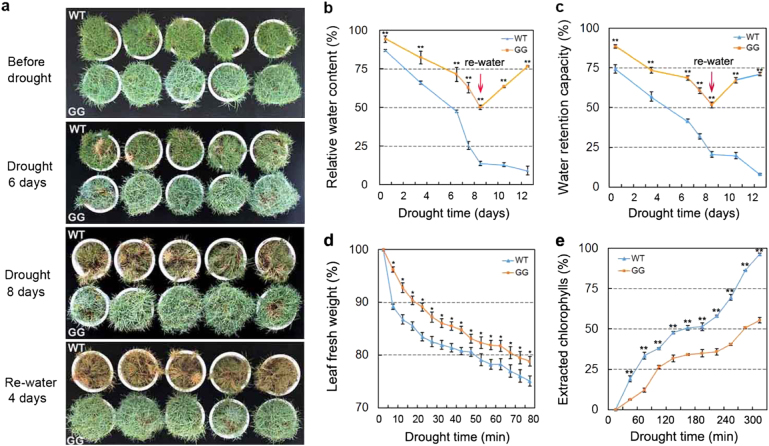
Drought tolerance assay of WT and GG *D. spiculifolius* plants. **a** Morphological appearance of WT and GG under normal and drought stress. Three-month-old plants were drought stressed (30 °C) for 8 days and rewatered for 4 days. Comparison of relative water content (**b**) and water retention capacity (**c**) of normal and stress-treated plants. Water loss (**d**) and chlorophyll leaching assays (**e**) of WT and GG under drought stress. The leaves of plants acclimated to darkness for 12 h to assure stomatal closure were subjected to water loss measurements and chlorophyll leaching assays. The leaves were weighed at the indicated time points or soaked in 80% ethanol for the indicated time points. Extracted chlorophyll contents at individual time points are expressed as percentages of that at 6 h after initial immersion. Asterisks indicate significant differences between WT and GG plants (*P < 0.05; **P < 0.01; Student’s *t* test). Error bars represent SE (*n* = 6)

Cuticular wax accumulation is reportedly associated with drought tolerance in plants^6,8^. Therefore, we investigated the drought tolerance of GG and WT plants. Under drought stress, GG plants exhibited a clear resistant phenotype, as indicated by their morphological appearance (i.e., less leaf wilting and death during the drought treatment) (Fig. 3a). GG leaves exhibited significantly higher RWC and water holding capacity than did WT leaves under both normal and drought-stress conditions (Fig. 3b, c). This result indicated that GG plants had higher drought resistance than did WT plants. Further, to determine whether cuticular wax accumulation is directly related to drought resistance, we measured cuticular transpiration and chlorophyll leaching of GG and WT plants under drought stress. Cuticular transpiration (Fig. 3d) and chlorophyll extraction (Fig. 3e) occurred more slowly in drought-treated GG leaves than in WT leaves, certainly due to cuticular wax accumulation. These results indicate that cuticular wax accumulation in GG leaves is linked with their drought resistance response.

### **Transcriptome sequencing of*D. spiculifolius*cuticular wax mutant leaves**

To identify the regulatory genes involved in wax biosynthesis in GG leaves, we performed transcriptome sequencing of GG and WT leaves. In total, approximately 51–52 million raw reads were obtained. After data cleaning, we obtained 37 641 222 and 38 394 140 clean reads for the GG and WT leaf samples, respectively. After clustering high-quality reads, 57 519 unigenes with an average length of 894 bp were obtained for the two samples. Using sequence- and domain-based alignments, sequence similarity searches were performed to validate and annotate the assembled unigenes. In total, 37 097 (64.50% of all unigenes), 20 064 (34.88%), 24 087 (41.88%), 27 206 (47.30%), 28 649 (49.81%), 30 689 (53.35%), and 19 018 (33.06%) unigenes were found in the NR, NT, SwissProt, KEGG, KOG, InterPro, and GO databases, respectively (Table 1). Overall, 39 278 genes (68.29% of all unigenes) were annotated in the aforementioned databases. The percentage is similar to that in the near-source species *Dianthus caryophyllus* ^24^.

**Table 1:** Number of functional annotations for all of the unigenes in public databases

**Annotated Database**	**Number of Unigenes**	**Percentage (%)**
NR	37,097	64.50%
NT	20,064	34.88%
SwissProt	24,087	41.88%
KEGG	27,206	47.30%
KOG	28,649	49.81%
InterPro	30,689	53.35%
GO	19,018	33.06%

### Comparison of gene expression levels in the cuticular wax mutant and wild type

**Fig. 4:**
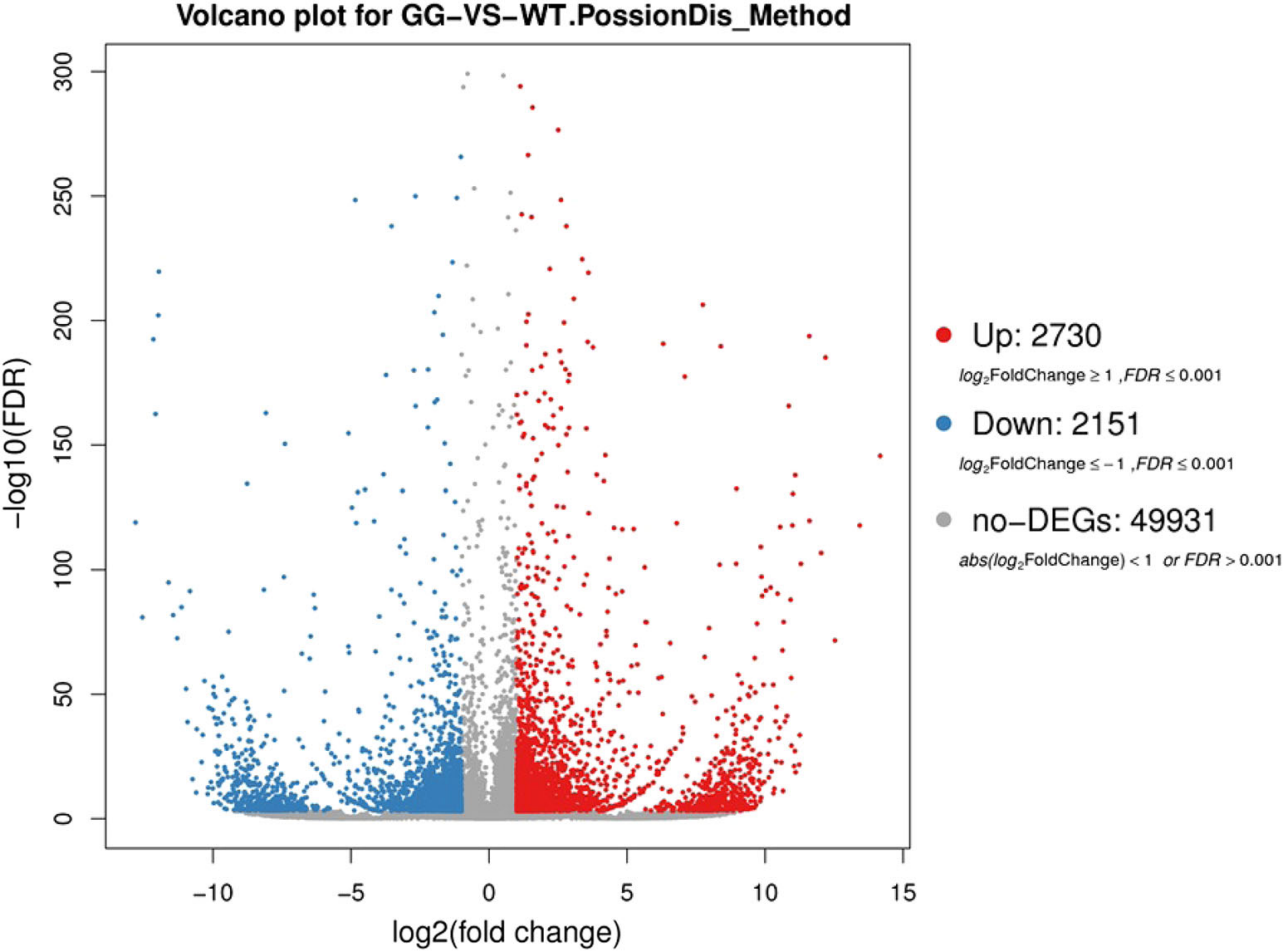
Volcano plots showing the number of differentially expressed genes (DEGs) in GG vs. WT *D. spiculifolius* plants. Abundance of each gene was normalized as RPKM. DEGs are shown in red (upregulated) and blue (downregulated), while gray indicates genes that were not differentially expressed (no-DEGs). We used a false discovery rate ≤ 0.05 and the absolute value of log_2_Ratio ≥ 1 as the threshold to judge the significance of the differences in gene expression

Using the RPKM method, the expression levels of the unigenes in GG plants to those in WT plants was compared. As shown in Fig. 4, a total of 4881 DEGs were identified, including 2730 upregulated and 2151 downregulated genes in GG leaf samples. Of these, 2228 upregulated and 1674 downregulated DEGs were ultimately annotated, but the functions of 979 genes could not be annotated (Supplementary Table S2).

### Functional classification and pathway enrichment analysis of DEGs

Using the GO annotation, the possible functions of the DEG were classified. The 2228 upregulated and 1674 downregulated DEGs were assigned to 46 and 47 functional groups, respectively; the three main categories were biological processes, cellular components, and molecular functions (Supplementary Figure S2).

**Fig. 5:**
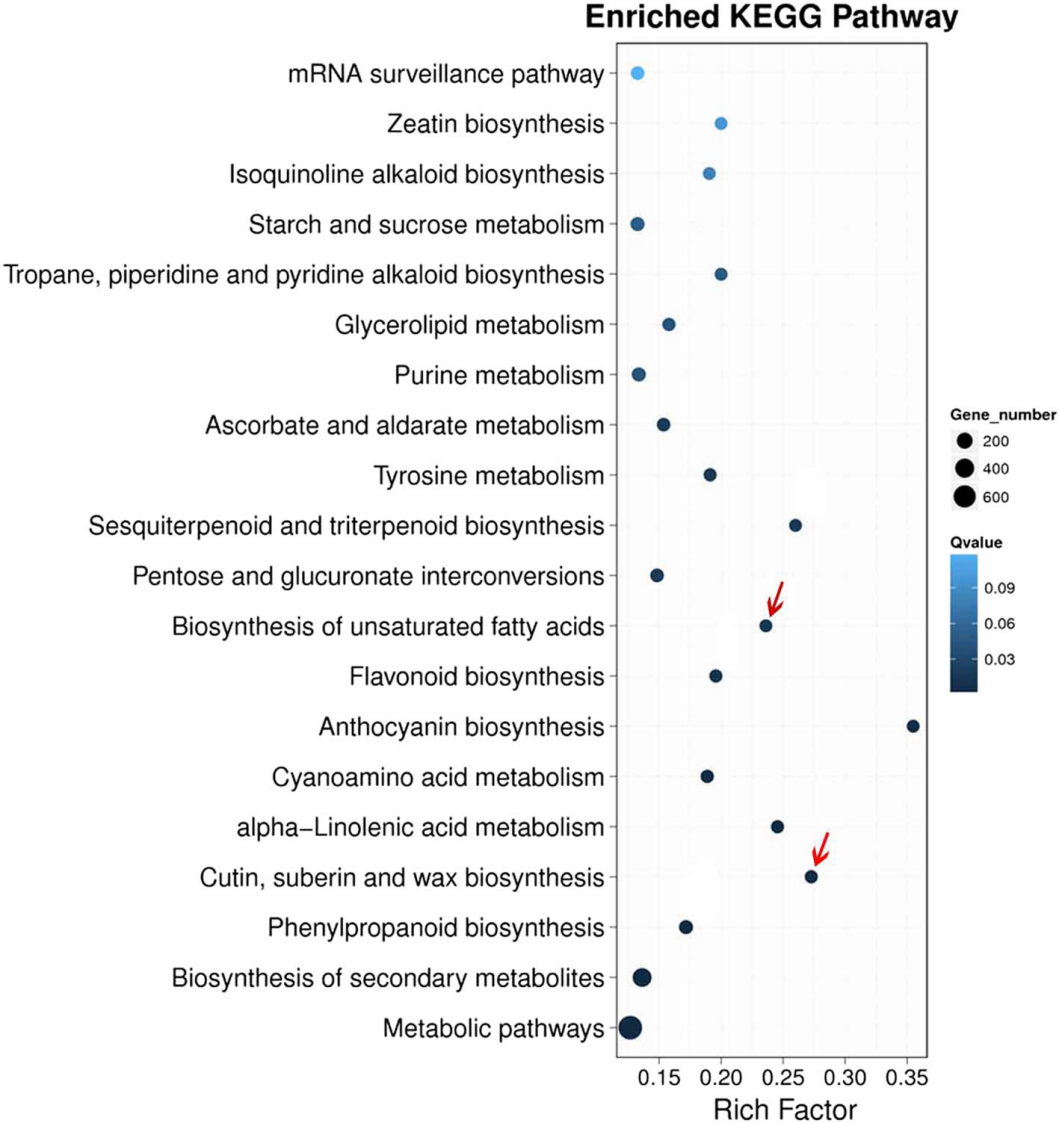
Scatterplot of KEGG pathways enriched for differentially expressed genes (DEGs) in GG vs. WT D*. spiculifolius* plants. The rich factor is the ratio of the number of DEGs annotated in a given pathway term to the number of all genes annotated in the pathway term. A greater rich factor means greater intensity. The *Q* value is the corrected *P* value and ranges from 0 to 1, and a lower *Q* value indicates greater intensity. The size of the circles indicates the number of genes. The top 20 enriched pathway terms in the KEGG database are listed. The red arrow indicates the significantly enriched pathway associated with wax biosynthesis

To further understand the functions of the DEGs, KEGG pathway enrichment analysis involving DEGs was conducted by mapping to KEGG database. The top 20 metabolic pathways associated with the DEGs (P ≤ 0.05) are shown in Fig. 5. Among these, metabolism and biosynthesis pathways of secondary metabolites were the most abundant. However, the rich factor of DEGs involved in biosynthetic pathways for anthocyanin, cutin, suberin and wax, and unsaturated fatty acids was highest. These results suggest that DEGs involved in wax and unsaturated fatty acid biosynthesis may play an important role in the cuticular wax accumulation on the leaves of GG plants. Among all DEGs evaluated, the number of MYB and MYB-related transcription factors was the largest (Supplementary Figure S3). Previous studies have shown that the R2R3-type MYB transcription factors act as transcriptional activators of genes involved in cuticular wax biosynthesis by binding conserved motifs in the gene promoters^5^.

### Analysis and qPCR validation of genes involved in wax biosynthesis

**Fig. 6:**
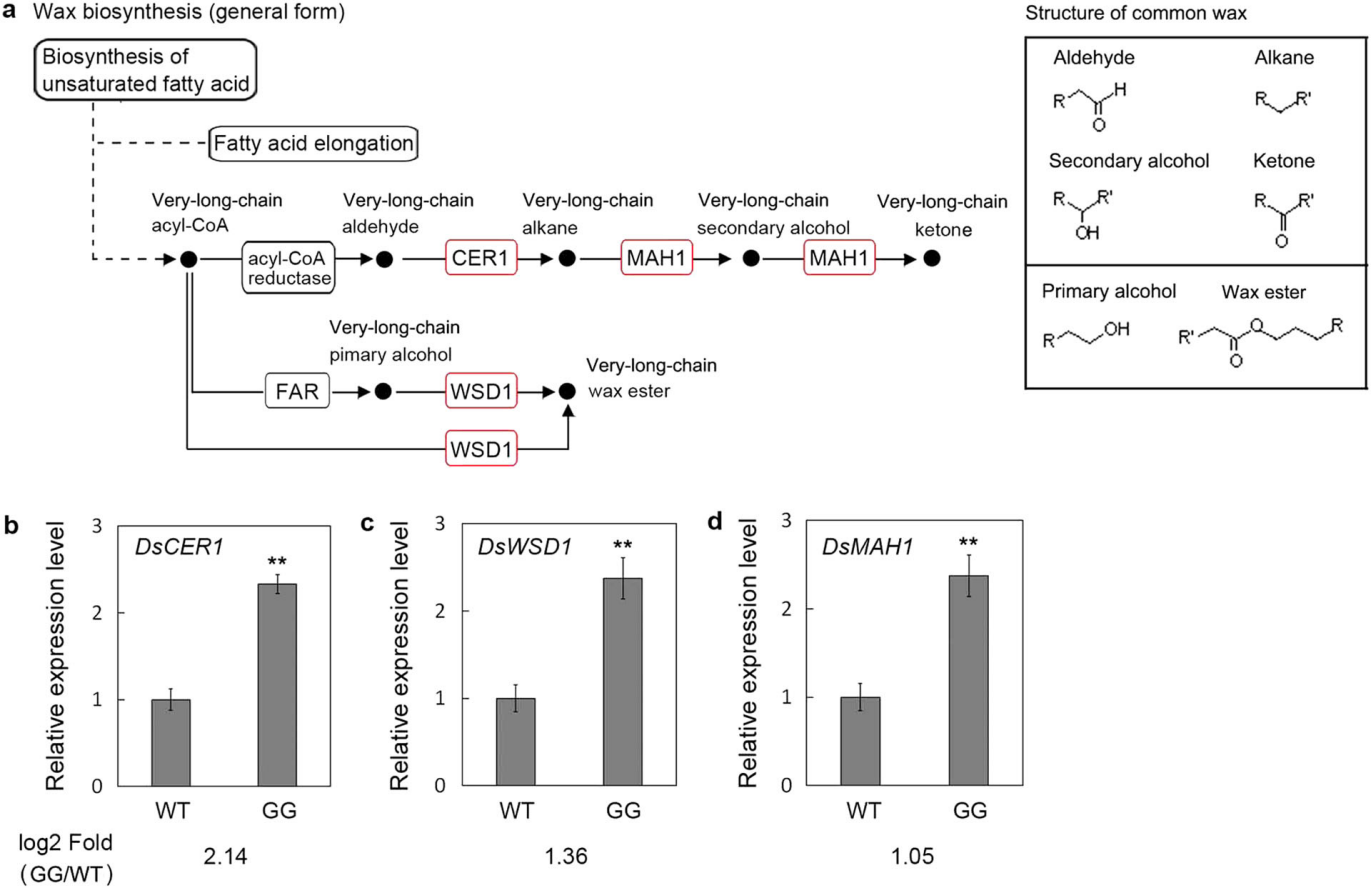
qPCR verification of key genes involved in the wax biosynthesis pathway in *D. spiculifolius*. **a** Simplified cuticular wax biosynthetic pathway from the KEGG database. Upregulated genes in GG vs. WT *D. spiculifolius* plants are marked with red borders. The structural information about the wax compounds is displayed in the black box. **b**–**d** qPCR analysis of key genes (*DsCER1*, *DsWSD1*, and *DsMAH1*) involved in wax biosynthesis. The *DsActin* gene was used as an internal control, and the transcript level in WT plants was set as 1.0. Asterisks indicate significant differences between WT and GG plants (**P < 0.01; Student’s *t* test). Error bars represent SE (*n* = 3)

RNA-seq results showed that three genes, *DsCER1*, *DsWSD1*, and *DsMAH1*, which were all involved in wax biosynthesis pathways, were upregulated in GG leaves (Fig. 6a). To evaluate the validity of RNA-seq, the expression levels of *DsCER1*, *DsWSD1*, and *DsMAH1* were evaluated by qPCR. The three genes exhibited differential expression in GG and WT leaves. The results of the qPCR assay were generally in agreement with the RNA-seq data (evaluated by RPKM) (Fig. 6b–d), confirming the reproducibility of the RNA-seq results.

## Discussion

The GG mutant of *D. spiculifolius* was first discovered in the M2 generation of EMS-mutagenized plants by leaf color (Fig. 1a). Plant leaf color is closely related to pigment content, and analysis of GG leaves showed that their chlorophyll and carotenoid contents were significantly higher than those in WT plants (Fig. 1d, e). Therefore, we speculated that leaf surface substances in GG plants may differ from those in WT plants. Further, the leaf surfaces of GG and WT plants were observed by SEM and cryo-SEM, which showed that the wax crystals on the leaf surfaces of GG plants were significantly more abundant than those on WT plants (Fig. 2a). Thus, we determined that the *D. spiculifolius* GG mutant is a high-cuticular-wax mutant. The change in leaf color of GG plants is due to high cuticular wax accumulation. Studies have shown that cuticular wax accumulation is related to drought resistance in plants^6,8^. As expected, the GG mutant showed a clear resistant phenotype when under drought stress, as indicated by its morphological appearance, RWC, and water holding capacity (Fig. 3a–c). Stomatal density and aperture in GG leaves were similar to those in WT leaves (Fig. 2g, h), but cuticular transpiration assays showed that the rate of non-stomatal water loss in drought-treated GG leaves was slower than that in WT leaves (Fig. 3d). Moreover, chlorophyll leaching assays showed that chlorophyll was extracted more slowly from drought-treated GG leaves (Fig. 3e). These results further confirmed that cuticular wax accumulation in GG leaves is directly linked to the drought resistance response. Overall, our results suggest that high cuticular wax accumulation leads to strong drought tolerance in GG plants.

Plant wax biosynthesis is regulated by a series of genes^14^. We used transcriptome sequencing to screen genes related to wax biosynthesis in GG plants. DEG analysis showed that 2 730 and 2151 up- and downregulated genes, respectively, were identified in GG leaves (Fig. 4). KEGG pathway enrichment analysis showed that the cutin, suberin, and wax biosynthesis pathway was significantly enriched (Fig. 5), and RNA-seq results showed that the *DsCER1*, *DsWSD1*, and *DsMAH1* genes were up-regulated in this pathway (Fig. 6a). Furthermore, the expression of these three genes was verified by qPCR, the results of which were essentially consistent with those of the RNA-seq analysis (Fig. 6b–d). Studies have shown that these three genes are the key genes regulating the biosynthesis of cuticular wax in plants^15–17^. These results suggest that changes in the expression of wax biosynthesis genes may affect the biosynthesis of cuticular wax in the leaves of GG plants. It has been observed that MYB is an upstream regulator of cuticular wax biosynthesis genes, and affects plant drought resistance by regulating cuticular wax accumulation^5,25,26^. 18 MYB and 16 MYB-related transcription factors were identified in all DEGs (Figure S3). This result suggests that the differential expression of these MYB transcription factors may be related to the up-regulation of wax biosynthesis genes. In addition, the anthocyanin biosynthesis pathway is also significantly enriched (Fig. 5), and we cannot rule out that the genes involved in this pathway also play a role in the phenotypic formation and drought resistance of GG plants. Furthermore, high chlorophyll and carotenoid content (Fig. 1d,e) may also play a role in the drought resistance of GG plants. However, the mechanisms up-regulating chlorophyll and carotenoid content in the leaves of GG plants warrant further investigation. In summary, our results showed that differential expression of wax biosynthesis genes in the leaves of GG plants resulted in an increase in cuticular wax and thus increased drought resistance.

## Electronic supplementary material

Supplementary Figures

Supplementary table S1

Supplementary table S2
