# Supplementary material for: Characterization of increased cuticular wax mutant and analysis of genes involved in wax biosynthesis in Dianthus spiculifolius
Source: Hortic Res. 2018 Aug 1;5:40. doi: 10.1038/s41438-018-0044-z (PMC6068182; doi:10.1038/s41438-018-0044-z)
Supplement: Supplementary file 1 — Supplementary Figures [file 41438_2018_44_MOESM1_ESM.pdf]

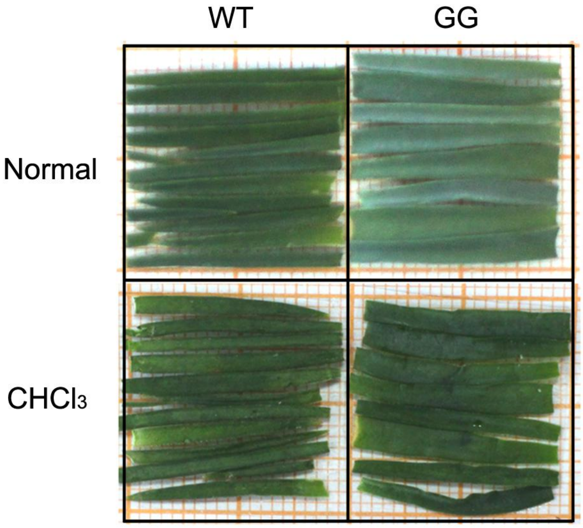

Figure S1. Normal and chloroform-treated leaves from WT and GG *D. spiculifolius* plants

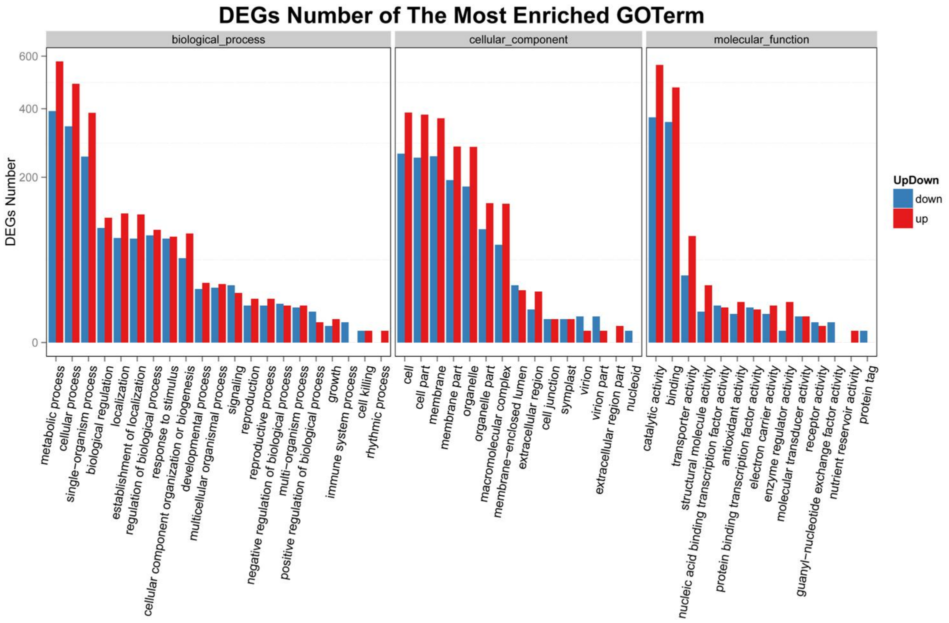

Figure S2. Histogram of gene ontology (GO) classification for differentially expressed genes (DEGs) in cuticular wax mutant (GG) vs. wild type (WT) *Dianthus spiculifolius* plants. Gene Ontology (GO) classification of up- (red) and down-regulated (blue) DEGs. Results are summarized in three main categories: biological processes, cellular components, and molecular function.

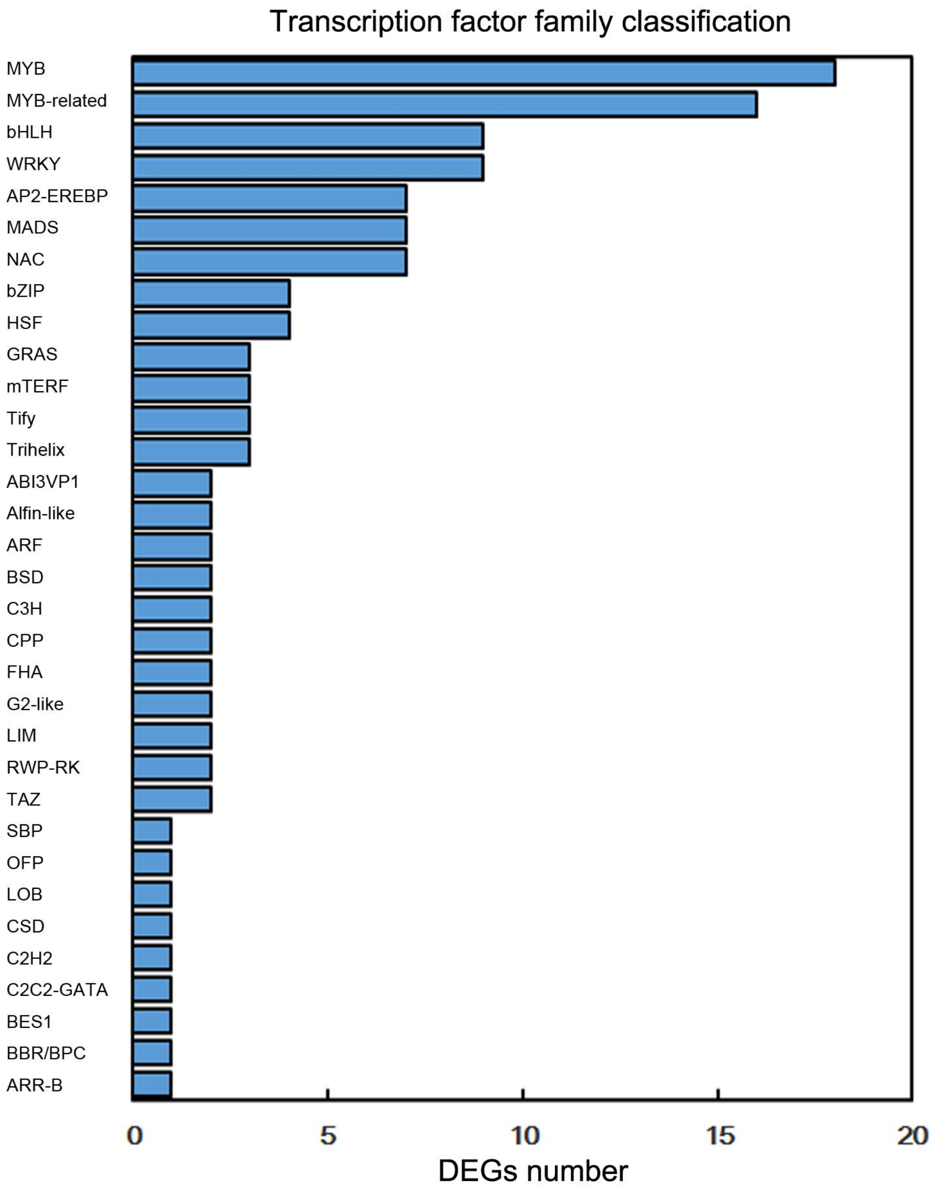

Figure S3. Transcription factor family classification for differentially expressed genes (DEGs) in cuticular wax mutant (GG) vs. wild type (WT) *Dianthus spiculifolius* plants. Transcription factor family is on the y-axis.
